# Impact of high-access exercise prior to and during early adolescence on later vulnerability to opioid use and relapse in male rats

**DOI:** 10.1038/s41398-022-02180-w

**Published:** 2022-10-03

**Authors:** Wendy J. Lynch, Anousheh Bakhti-Suroosh, Jean M. Abel

**Affiliations:** Department of Psychiatry and Neurobehavioral Sciences, University of Virginia, Charlottesville, VA 22904 USA

**Keywords:** Addiction, Diseases, Neuroscience

## Abstract

Middle- and high-school athletes participating in certain team sports are at greater risk of opioid misuse and addiction than those who do not. While this risk is thought to be due to increased access to opioids, in this study we explored the possibility that the sensitizing effects of discontinued high-intensity exercise may also contribute. Specifically, using male rat models with fentanyl, we tested the hypothesis that high-access exercise (24 h/day access to a running wheel) during pre/early adolescence (two weeks, postnatal-day 24–37) would enhance vulnerability to opioid use and relapse during late adolescence/adulthood. Rats with a history of high-access exercise showed stronger fentanyl-associated lever discrimination during acquisition, greater motivation to obtain infusions of fentanyl following acquisition, and had an enhanced sensitivity to the reinstating effects of fentanyl-associated cues following extended (24 h/day), intermittent-access self-administration and protracted abstinence (14 days) compared to sedentary controls. In contrast, sedentary rats had greater overall responding (active- and inactive-lever) during acquisition and greater non-specific (inactive-lever) responding during extended-access self-administration. Molecular markers associated with opioid seeking/relapse were also differentially expressed in the nucleus accumbens core of rats with versus without a history of high-access exercise following relapse testing (e.g., *Bdnf-IV* and *Drd2* expression). Together, these findings demonstrate that high-access exercise prior to and throughout early-adolescence enhances vulnerability to the reinforcing and cue-induced reinstating effects of opioids during later adolescence/adulthood. Thus, it is possible that the discontinuation of high intensity exercise contributes to the enhanced vulnerability observed in middle- and high-school athletes.

## Introduction

Opioid use and addiction (or opioid use disorder, OUD) is a major epidemic in the United States, and in the past year alone (12-month period ending April 2021), accounted for over 100,000 deaths with synthetic opioids, such as fentanyl, being a major culprit [1]. Effects of this epidemic are widespread, and adolescents and young adults are at high risk considering that rates of opioid-induced overdose deaths have tripled in individuals under the age of 20 in the last two decades; [2] adolescents and young adults also have an accelerated time-course for the development of addiction compared to adults [3]. This risk is further exacerbated among middle- and high-school athletes participating in sports associated with a high risk of injury (e.g., football, wrestling) given that these populations have a 50% higher odds of prescription opioid misuse and an increased risk of heroin use compared to their peers not participating in these types of sports [4, 5]. Participation in contact-sports during high-school also predicts adult prescription opioid misuse and OUD [6].

The increased risk is believed to be due to increased access to opioids in these populations (i.e., for sport-related injuries; [7]). Indeed, numerous awareness campaigns and intervention efforts have been implemented across the country to highlight the risk of prescription opioid use in kids following sports injury (e.g., Billboard campaign, “*Don’t Let Your Athlete Become Addicted, Treating injuries with prescription pain killers can lead to heroin addiction”*, www.drugfree.org; ref. [8]). However, an unexplored hypothesis is that the enhanced vulnerability occurs as a result of discontinued high-intensity exercise, which based on preclinical findings with other addictive drugs, can sensitize the reward pathway and enhance sensitivity to the rewarding/reinforcing effects of drugs; [9–12] also see [13, 14]. Specifically, while continued daily exercise at high or low intensities (e.g., 2 to 22 h/day access to a wheel; [13–19]) or discontinued exercise at low intensity decreases the rewarding/reinforcing effects of addictive drugs (e.g., treadmill running up to 60 min/day [20]), chronic (≥ 2 weeks) high intensity exercise (e.g., 24 h/day access to a running wheel) that is discontinued prior to drug exposure enhances the rewarding effects of addictive drugs, including opioids, as assessed using conditioned place preference [9, 11, 12]. Results with methamphetamine further show that rats with a history of high-access exercise (24 h/day-access to a wheel, 6 weeks) acquired drug self-administration faster and were more motivated to obtain infusions of the drug following extended-access (6 h/day) self-administration as compared to sedentary controls [10]. High-access exercise also induces long-term changes in neuroplasticity reflected by gene expression changes targeting brain-derived neurotropic factor (BDNF), dopamine, and opioid receptors in key regions of the reward pathway (e.g., nucleus accumbens, NAc; [21–23]). Many of these molecular changes also occur following opioid exposure/use with the development of addiction-like features (e.g., enhanced vulnerability to relapse; [24–27]), indicating that high-access exercise may “prime” the mesolimbic pathway for later opioid use and OUD.

This central hypothesis was tested in this study using rat models with fentanyl. Fentanyl is highly potent opioid (~100 times more potent than morphine), and like morphine and other highly prescribed and misused opioids, it has a high affinity and selectivity for mu opioid receptors over delta and kappa receptors [28]. The National Institute on Drug Abuse identified studies targeting fentanyl specifically as a major research initiative [29], yet fentanyl is rarely used in preclinical models of OUD. Based on results showing that risk of opioid misuse emerges in boys during middle-school [4], we examined the effects of high-access exercise during pre/early adolescence (postnatal-day 24–37) on vulnerability to opioid use and relapse during late adolescence/adulthood (postnatal-day 40–80+) in male rats. High-intensity exercise was modeled using an unlimited, 24 h/day voluntary wheel running procedure (two weeks of continuous access throughout pre/early adolescence) and later vulnerability to opioid use was as assessed by rates of acquisition of fentanyl self-administration and motivation to obtain fentanyl as defined by breakpoints reached under a progressive-ratio (PR) schedule. We also determined effects on vulnerability to relapse, as assessed by levels of drug-seeking during extinction and cue-induced reinstatement testing. Effects were determined following extended (24 h/day), intermittent-access fentanyl self-administration and protracted abstinence (14-days) since these conditions are known to induce high levels of fentanyl-seeking [30]. Relapse-associated neuroadaptations were also examined compared between rats with and without a history of high-access exercise focusing on markers implicated in opioid use/relapse (Bdnf exon IV, *Bdnf-IV*; dopamine receptor 1, 2, 3, *Drd1, Drd2, Drd3*; mu opioid receptor 1, *Opmr1;* [26, 27, 31–33]. We also previously showed that continued moderate exercise that decreased opioid self-administration (resistance exercise, ~1 h/day; [33] increased NAc *Bdnf-IV* and decreased NAc *Oprm1, Drd1, Drd2, and Drd3*. We hypothesized that relative to sedentary controls, rats with a history of high-access exercise would acquire fentanyl self-administration more rapidly, be more motivated to obtain infusions of fentanyl, and show greater cue-induced fentanyl-seeking and differential gene expression changes following extended-access self-administration and protracted abstinence.

## Materials and methods

### Subjects

Male (*N* = 35) Sprague Dawley rats (Charles River Laboratories) arrived at postnatal 22 and were singly-housed in polycarbonate cages with a 35.6 cm diameter running wheel attached (ENV-046; Med-Associates); a metal gate that separated the polycarbonate cage from the wheel was kept in place until the exercise/sedentary sessions began as described below. Rats were maintained on a 12 h light/dark cycle (lights on at 7-AM) and had *ad libitum* access to food and water throughout the study. Body weights were determined three times each week and health was monitored daily. All procedures were approved by the University of Virginia Animal Care and Use Committee and were conducted in accordance with NIH guidelines.

### Procedures

#### High-access exercise during pre/early adolescence

**Fig. 1:**
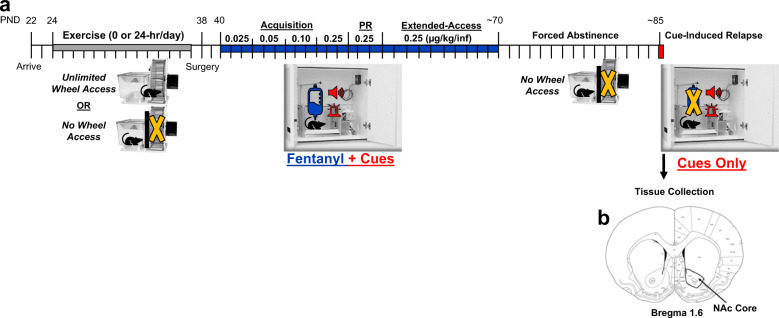
Experimental timeline. **a** Rats arrived on postnatal-day (PND) 22 and were given high-access exercise (24-hr/day) or no wheel access for 2-weeks (PND 24–37). Following recovery from catheterization surgery, rats underwent acquisition testing under an escalating dose procedure (0.025, 0.05, 0.1, 0.25 µg/kg/infusion, 3 days/dose; ~PND 40–51). PR testing for fentanyl (0.25 µg/kg/infusion) began after the final acquisition test session (~PND52). Rats were then given extended, 24 h/day, access to fentanyl for 10 days using an intermittent-access procedure (fixed-ratio 1, 5 min trials every 30 min; PND ~58–68). Vulnerability to relapse was assessed following a 14-day abstinence period using a within-session extinction/cue-induced reinstatement procedure. Gray and blue bars show periods of exercise and fentanyl availability, respectively. **b** Tissue was dissected from the core region of the nucleus accumbens (NAc) the day following the relapse test session.

Rats were randomly assigned to an exercise (*n* = 13) or sedentary (*n* = 12) group. Exercise/sedentary sessions began after a two-day habituation period on postnatal-day 24 (Fig. 1a). The exercise session was initiated by removing the gate that separated the polycarbonate cage from the wheel; rats then had 24 h/day-access to the unlocked running wheel for 14 days (until postnatal-day 37). This level and length of exercise access was selected based on previous results showing that levels of running increase to high levels under these conditions [34]; importantly, discontinued exercise at this level (24 h/day-access for two or more weeks) enhances later sensitivity to the rewarding/reinforcing effects of addictive drugs, including opioids (e.g., morphine; [9–12]). This time-line of unlimited access exercise also corresponds to pre/early adolescence, which is known to be a critical developmental period of heightened brain malleability [35]. The exercise period ended following the 14th session wherein the gate was reintroduced to prevent further wheel access. Wheel revolutions were recorded for each 24 h period. Sedentary rats underwent similar handling, but because a locked wheel can serve as a modified exercise condition [16], the gate remained in place prohibiting access to the wheel.

#### Catheter implantation and maintenance

Rats were implanted with a right jugular vein catheter on postnatal-day 38 as previously described [17], and once recovered, they were singly-housed in operant chambers (ENV-018M, Med-Associates). Catheters were flushed with heparinized saline three times each week; patency was verified weekly during acquisition and periodically thereafter using methohexital (1.5 mg/kg, IV). One rat in the exercise group lost patency during acquisition and was excluded from the study (final *n* = 12). Patency loss after acquisition was common considering that these adolescent rats doubled in size from the start to the end of the self-administration phase (from ~200 to ~400 g), and in these cases, the rat was implanted with a new catheter in the left jugular vein with testing resuming after 1–2 days.

#### Acquisition of fentanyl self-administration

Acquisition testing began on postnatal-day 40 using an escalating dose procedure. With this procedure, rats had access to one of four doses of fentanyl during daily sessions beginning with three sessions at 0.025 µg/kg/infusion, then 3 sessions at 0.05 µg/kg/infusion, 3 sessions at 0.1 µg/kg/infusion, and finally three or four sessions at 0.25 µg/kg/infusion. This dosing regimen was selected to both maximize individual differences and ensure that the majority of rats would acquire by the end of the testing period [30, 36–38]. Sessions began at 12-PM with the extension of the active- (left) lever into the operant chamber and the delivery of one fentanyl infusion. Active-lever responses were reinforced under a fixed-ratio 1 schedule and each infusion was paired with a stimulus light located above the active-lever and the sound of the pump (PHM-100, Med-Associates) which was adjacent to the operant chamber within the sound-attenuating box. The active-lever remained extended until all 40 infusions were obtained or until 9-AM the next day (up to 21 h). The inactive- (right) lever was extended into the operant chamber throughout the study; responses were recorded but were without consequence. As with previous studies, acquisition was defined based on intake (three consecutive sessions wherein all 40 available infusions were obtained) and a preference for the active- versus inactive-lever (≤60%; e.g., [39–41]); the first session of the three was designated as the acquisition day. While the majority of the rats tested met the infusion criterion within 12 days, 2 rats in the exercise group and one rat in the sedentary group required one additional session to meet this criterion. One rat in the exercise group did not meet the infusion criterion within 13 sessions and was designated as “did not acquire” and was not further tested. Another rat in this group had to be excluded after acquisition due to health issues. Three rats in the sedentary group met the infusion criterion, but not the lever preference criterion within 13 test sessions; these rats were also designated as “did not acquire” for acquisition, but since they met the infusion criterion, they moved forward to PR and extended-access testing. All three rats acquired the active-lever discrimination by day 1 of extended-access; their data were included for extended-access, but not PR.

#### PR fentanyl self-administration

Following acquisition, rats with (*n* = 10) and without (*n* = 9) a history of exercise were tested on motivation for fentanyl (0.25 µg/kg/infusion) using a PR schedule [42]. PR sessions began at approximately postnatal-day 53 and were conducted as described for acquisition testing except that the number of responses required to obtain an infusion increased throughout each session in the following steps: 1, 2, 4, 6, 9, 12, 15, 20, 25, 32, 40, 50, etc; ref. [43]. there was also no limit on infusions and the active-lever remained extended until 1 h before the next PR session (11 AM). Sessions continued daily until a stable baseline was obtained (no increasing or decreasing trend in the number of infusions obtained over three consecutive sessions; typically 3–4 sessions). PR data from one sedentary rat were excluded due to patency/health issues (final *n* = 8).

#### Extended-access fentanyl self-administration

Following the last PR session, rats with (*n* = 10) and without (*n* = 11) a history of exercise were given extended- (24 h/day) intermittent-access to fentanyl [30]. Since patency/health issues during extended-access prevented the inclusion of three rats in the history of exercise group and one rat in the sedentary group, we included additional rats with (*n* = 5) and without (*n* = 5) a history of exercise that underwent the same exercise/sedentary and surgical conditions as described above. However, in order to maximize acquisition, these rats were trained to self-administer to the highest fentanyl dose (0.25 µg/kg infusions) over five to ten sessions until acquisition occurred. While these rats were slightly younger at the start of the extended-access phase as compared to those tested under the escalating-dose acquisition procedure (postnatal-day 48 ± 1 versus 61 ± 1), their extended-access data were indistinguishable from the escalating-dose group for both intake (60 ± 4 versus 58 ± 19 µg/kg/day) and responses (active-lever, 358 ± 33 versus 346 ± 37; inactive-lever, 58 ± 9 versus 41 ± 9) averaged across the 10-day period; there were also no significant overall or interactive effects of cohort (see “Data Analysis”). Thus, these data were included for a final *n* of 12 and 15 for rats with, and without, a history of exercise, respectively in order to provide adequate power for detecting group differences.

Our extended-access procedure was adapted from a procedure developed for cocaine that readily induces the development of addiction-like features [44], including an enhanced vulnerability to relapse; [45] also see [46]. With this procedure, rats had fixed-ratio 1 access to rapidly delivered (~1 s) infusions of fentanyl (0.25 mg/kg/infusion) in 5 min trials that initiated every 30 min 24 h/day for ten days [30]. Extended-access sessions began at approximately postnatal-day 55. Each trial began with the extension of the active-lever into the chamber; no priming infusions were administered and there were no limits on infusions per trial or day. The active-lever retracted at the end of each trial. Rats were moved to a polycarbonate cage without wheel access following the last extended-access session.

#### Reinstatement of fentanyl-seeking

Vulnerability to relapse was assessed in a subset of rats in the history of exercise (*n* = 8) and sedentary (*n* = 10) groups following extended-access self-administration and 14 days of abstinence. Rats were placed back into their operant chambers on abstinence day 14 to re-habituate them to their chambers overnight. Fentanyl-seeking was assessed the next day using a within-session extinction/cue-induced reinstatement procedure [30]. Extinction responding was assessed in 6–9, 1 h sessions wherein responses were recorded, but did not have a consequence. Cue-induced reinstatement of responding was examined once responding extinguished (≤15 responses/session). One non-contingent administration of the cues formerly associated with fentanyl (sound of pump and stimulus light above the active-lever, ~1 s) was administered at the start of the 1 h session; each response on the formerly active-lever produced these cues.

#### Gene expression

Relapse-associated changes in gene expression were examined in the NAc of a subset of rats in the history of exercise (*n* = 7) and sedentary (*n* = 7) groups. Rats were anesthetized with isoflurane and then euthanized by rapid decapitation the morning following the extinction/cue-induced reinstatement test. Tissue from the NAc core was dissected from coronal brain slices (see Fig. 1b), rapidly frozen, and stored at −80 °C [33]. Total RNA was isolated using the RNeasy® Lipid Tissue Mini Kit (Qiagen, Valencia, CA), the quantity and quality of the RNA was assessed using the NanoVue™ Spectrophotometer, cDNA templates were prepared using the High-Capacity cDNA Reverse Transcription Kit (Applied Biosystems, Carlsbad, CA), and q-PCR was performed using the ABI StepOnePlus real-time PCR system [33]. Applied Biosystems TaqMan™ Gene Expression assays (Drd-2, Rn00561126; Oprm1, Rn01430371) and SYBR™ Green-Based Detection (Invitrogen primers: *Bdnf-IV*, *Drd-1*/3, *Gapdh* [47, 48]). Samples were normalized to Gapdh (Rn01775763). Genes were measured in triplicate for each sample and real-time run to avoid inter-sample variance and each SYBR™Green-Based detection reaction was verified for a single PCR product of expected size with the disassociation melting curve stage. Genes were analyzed with StepOne™ software and summarized blind to experimental conditions [33]. One rat in the sedentary group was a major outlier for *Bdnf-IV* (based on a Grubb’s test); this data point was excluded from this analysis for a final *n* of 7 and 6 for the history of exercise and sedentary groups, respectively. The final *n* was 7 for both groups for all other genes.

### Drugs

Fentanyl hydrochloride was obtained from NIDA (Research Triangle Park, NC), dissolved in saline, sterile filtered, and stored at 4 °C. The infusion duration was adjusted three times/week based on body weight to ensure that the dose (mg/kg) remained constant throughout the study.

### Data analysis

Levels of running within each 24 h period were analyzed over the 2-week period using repeated measures ANOVA with additional comparisons made between the first and last five exercise sessions. A Meier survival analysis and Mantel–Cox rank statistic were used to compare rates and percent group acquisition between the groups [17]. Group differences in the two criteria used to define acquisition, number of infusions and active-lever discrimination were examined in separate mixed-effects models (restricted maximum likelihood, residual method). For infusions, group (history of exercise, sedentary), session [1–12], and their interaction were included as fixed-factors; for active-lever discrimination, lever (active, inactive) was included as an additional fixed-factor. Group differences in responses and intake during the 10-day extended-access period were similarly analyzed except that active- and inactive-lever responses were examined separately since their scales were markedly different and acquisition cohort was also included as a between-subject factor; however, given that no significant overall or interactive effects were observed, it was deleted from the model. Mixed effects analyses were also used to examine group differences body weights at the start and end of acquisition testing and the start and end of the extended-access period, infusions obtained over the three stable PR sessions, and responses during each extinction session (typically 6, but up to 8) and between the last extinction session and the reinstatement session. A chi-square test was used to determine group differences in the probability of reinstatement of fentanyl-seeking. Differences in gene expression were evaluated using Benjamini-Hochberg corrected t-tests. Associations between levels of prior exercise and effects during each of the phases (days to acquire, average PR infusions, average extended-access intake and responses, total extinction and reinstatement responses, gene expression) were analyzed using the Pearson Correlation Co-efficient focusing on measured that differed/tended to differ between groups. One-tailed t-tests were used for a priori predicted differences (higher motivation in the history of exercise group; escalation of responding for fentanyl during extended-access; increased *Drd1/2/3*, *Oprm1*; decreased *Bdnf-IV*); two-tailed tests were used for all other comparisons. All post hoc comparisons were Bonferroni-corrected. Statistical analyses were performed using SPSS.

## Results

### High-access exercise during adolescence

**Fig. 2:**
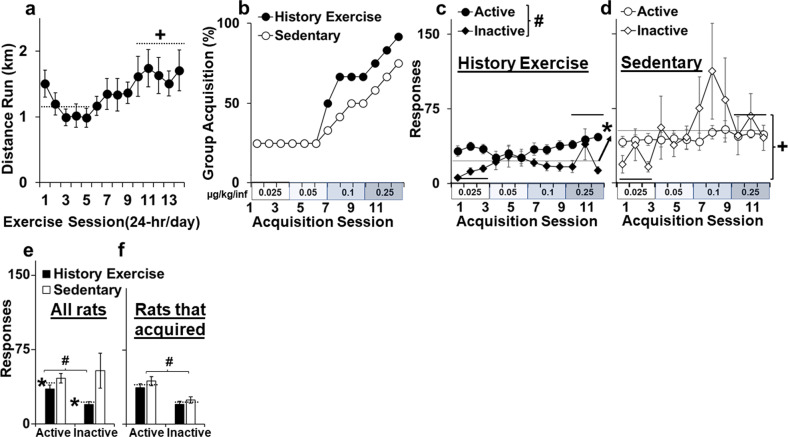
Effects of discontinued high-access exercise on acquisition of fentanyl self-administration. Average (± SEM) daily distance run in km for the history of exercise group (**a**) and percent group acquisition (**b**) and average (± SEM) active and inactive lever responses in the history of exercise (**c**) and sedentary (**d**) groups as a function of fentanyl dose/acquisition session (0.025, 0.05, 0.1, and 0.25 µg/kg/infusions for sessions 1–3, 4–6, 7–9, and 10–13, respectively). Average (± SEM) number of active- and inactive-lever responses across the acquisition testing period for all rats in the acquisition study (**e**) and just the rats that acquired fentanyl self-administration within the 12–13-day testing period (**f**). *Significant effect of group (P’s < 0.05; **b**–**d**); ^#^Significant effect of lever (P’s < 0.05; **b**, **e**, **d**-history of exercise); ^+^Significant difference between sessions 1–3 and 10–12 (*P* < 0.05; **b**, **c**).

Rats ran an average of 19 ± 2 km over the 2-week, 24 h/day high-access exercise period (Fig. 2a) Levels of running increased over time (effect of day, F_13,208_ = 3.2, *P* < 0.01; average distance over first five sessions versus last five sessions, *t*_16_ = 2.3, *P* < 0.05), and by the end of the 2-week period, rats were averaging 1.7 ± 0.3 km/day. Levels of running were also stable by the end of the 2-week period with results from the analysis of running within the first five sessions versus the last five sessions revealing a significant effect of day within the first five sessions only (F_13,208_ = 3.2, *P* < 0.01). There was considerable variability between subjects in levels of running, however, with average running distance over the two-week period ranging from 0.4 and 4.1 km/day.

### Acquisition of fentanyl self-administration

Acquisition rates were similar between rats with and without a history of high-access exercise (*P* > 0.05; Fig. 2b). In both groups, only a minority of rats acquired fentanyl self-administration within the first 6 days (25%); acquisition increased progressively thereafter, and by the end of the acquisition testing period, the majority of the history of exercise and sedentary rats had acquired self-administration (92% versus 75%, respectively). The number of days needed to acquire fentanyl self-administration was also similar between the history of exercise and sedentary groups (6.9 ± 1.3 and 7.0 ± 1.6, respectively, *P* > 0.05). Rats with and without a history of exercise weighed a similar amount at the beginning (203 ± 3 g versus 201 ± 4 g, respectively) and end of acquisition testing (304 ± 5 versus 303 ± 6 g, respectively; significant effect of day only, F_1,22_ = 1339, *P* < 0.001).

We also examined group differences for both criteria used to define acquisition, intake and active-lever discrimination in separate analyses. Although no difference was observed between the history of exercise and sedentary groups for intake (µg/kg/day) over acquisition (3.5 ± 0.2 and 3.9 ± 0.1, respectively, *P* > 0.05), a significant difference was observed for active-lever discrimination (group × lever, F_1,528_ = 11.7, *P* < 0.001; Fig. 2c–e), with post hoc comparison within each group revealing greater active than inactive responses in the history of exercise group (effect of lever, F_1,264_ = 47.0, *P* < 0.001), but not the sedentary group (effect of lever, *P* > 0.05). The analysis of active versus inactive responses also revealed significant effects of day (F_11,528_ = 2.2, *P* < 0.05), which reflects greater responding during the first three versus last three sessions (lowest versus highest dose; *P* < 0.01), and group (F_1,528_ = 4.7, *P* < 0.05). This latter effect reflects higher general activity (active and inactive responses) in the sedentary versus history of exercise group; post hoc comparison within each lever also revealed non-significant trends for higher active and inactive responses in the sedentary versus history of exercise group (*P* = 0.078 and 0.057, respectively). Importantly, among rats that acquired fentanyl self-administration within the 12- or 13-day testing period, active- and inactive-lever responding were similar between the sedentary and history of exercise groups (Fig. 2f). Thus, while rates and levels of acquisition were similar between groups, rats with, versus without, a history of high-access exercise showed greater active-lever discrimination but less overall responding indicating that the discontinuation of high-access exercise enhanced acquisition of fentanyl-lever discrimination but reduced fentanyl-induced locomotor activity.

### Motivation for fentanyl

**Fig. 3:**
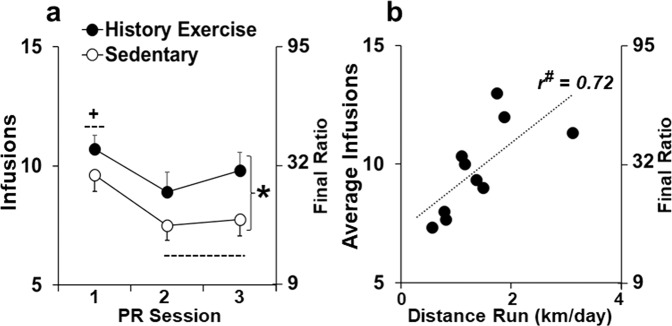
Effects of discontinued high-access exercise on motivation for fentanyl. **a** Number of infusions (± SEM) and corresponding final ratios reached under the progressive ratio (PR) schedule for each of the three stable sessions for the history of exercise and sedentary groups. **b** Average number of infusions (± SEM) and corresponding final ratios reached under the PR schedule as a function of average distance run/day over the 2-week high-access exercise for the history of exercise group. The Pearson correlation co-efficient (*r* value) for the relationship between infusions and distance run is also included. *Significant effect of group (*P* < 0.05; **a**); ^+^Significant difference between sessions 1 and 2–3 (*P* < 0.05; **a**). ^#^Significant association between infusions and distance run (*P* < 0.05; **b**).

Rats with, versus without, a history of high-access exercise obtained more fentanyl infusions over the three stable PR test sessions (Fig. 3a; F_1,48_ = 3.0, *P* < 0.05). This analysis also revealed a significant effect of session (F_2,48_ = 9.2, *P* < 0.001) with rats in both groups obtaining significantly more infusions on the first session (versus 2–3; *P* < 0.05). Inactive responses were similar between the history of exercise and sedentary groups (37 ± 10 and 32 ± 6 respectively). Within the history of exercise group, there was a significant correlation between average distance run during the high-access exercise period and number of infusions obtained over the three stable PR test sessions (Fig. 3b; *r*_10_ = 0.72, *P* < 0.05). Thus, rats with, versus without, a history of high-access exercise were more motivated to obtain fentanyl and higher levels of prior running were predictive of greater subsequent motivation.

### Extended-access fentanyl self-administration

**Fig. 4:**
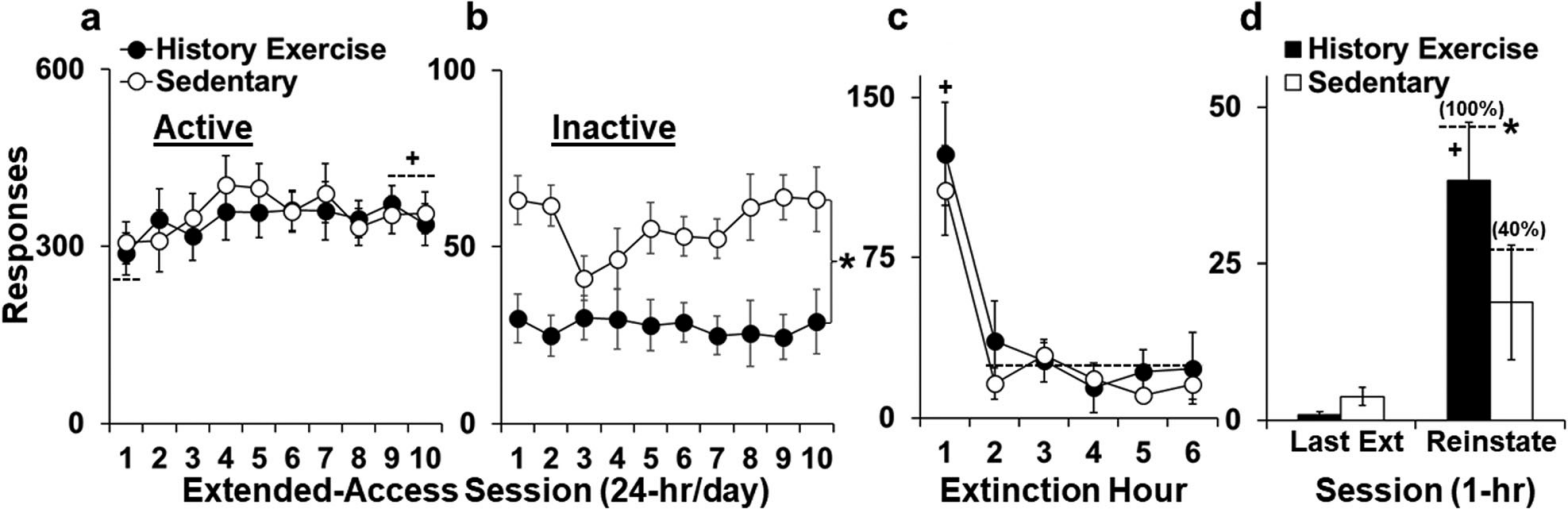
Effects of discontinued high-access exercise on extended-access fentanyl self-administration and subsequent relapse vulnerability. Number of active (**a**) and inactive (**b**) responses (± SEM) for each of the ten extended-access sessions for the history of exercise and sedentary groups. Number of responses (± SEM) for the first six extinction sessions run (**c**) and the last extinction session versus the reinstatement session (**d**) for the history of exercise and sedentary groups. The numbers in parenthesis are the percentage of rats within each group that showed reinstatement of responding (higher responses during reinstatement compared to the last extinction session). ^+^Significant difference between sessions 1 and 9–10 (*P* < 0.05; **a**), sessions 1 and 2–6 (*P* < 0.001; **c**), and the last extinction session versus reinstatement (*P* < 0.05, **d**-history of exercise). *Significant difference in percent group reinstatement (*P* < 0.05; **d**).

The history of exercise and sedentary groups self-administered similar levels of fentanyl over the 10-day extended-access period (61 ± 5 and 63 ± 6 µg/kg/day, respectively; *P* > 0.05). Active-lever responses were also similar between the groups although, as expected, responding escalated from the initial session to later ones (Fig. 4a; session 1 versus 9–10, *t*_26_ = 1.8, *P* < 0.05). In contrast, there was a robust group difference for inactive-lever responses with sedentary rats responding at higher levels than history of exercise rats (Fig. 4b; F_1,250_ = 6.9, *P* < 0.01) and this difference was similar across the 10 extended-access sessions (non-significant overall and interactive effects of day, *P*’s > 0.05). The history of exercise and sedentary groups weighed a similar amount at the start (329 ± 17 g and 329 ± 13 g, respectively) and end of extended-access self-administration (406 ± 19 and 403 ± 13 g, respectively; significant effect of time only, F_1,50_ = 101.4, *P* < 0.001). Within the exercise group, no significant association was observed for average distance run and inactive responses indicating that a history of high-access exercise, at any level, reduced subsequent sensitivity to fentanyl’s locomotor-enhancing effects.

### Vulnerability to relapse

The history of exercise and sedentary groups responded at similar levels during extinction (Fig. 4c; *P* > 0.05) and met the extinction criterion (≤15 responses/session) within a similar number of sessions (6.3 ± 0.2 and 6.5 ± 0.2, respectively; *P* > 0.05). They also showed a similar pattern of extinction responding with the highest responding occurring in both groups during the first session (effect of session, F_7,100_ = 14.0, *P* < 0.001; session 1 versus 2–6, *t*_17_ = 6.3, *P* < 0.001). In contrast, an effect of exercise history was evident during reinstatement testing such that responding was reinstated by fentanyl-associated cues in rats with, but not without, a history of exercise (Fig. 4d). In fact, responding was reinstated by the cues in all 8 history of exercise rats (e.g., higher responses in the reinstatement session than the last extinction session), compared to just 4 of the 10 sedentary rats (χ^2^ = 7.2, *P* < 0.01). A trend for a significant interaction of group by session was also observed in the mixed-effects analysis of responses during the last extinction session versus the reinstatement session (F_1,32_ = 3.6, *P* = 0.067) and follow-up comparisons within each group confirmed an effect of session within the history of exercise (*t*_7_ = 3.9, *P* < 0.01), but not sedentary group (*P* > 0.05). The history of exercise and sedentary groups responded at similarly low levels on the inactive-lever during extinction/reinstatement testing (2.6 ± 0.9 and 6.7 ± 2.3, respectively; *P* > 0.05) and weighed a similar amount on the test day (486 ± 21 g and 485 ± 14 g, respectively). Within the exercise group, no significant association was observed for average distance during the high-access exercise period and reinstatement responses indicating that a history of high-access exercise, at any level, enhances subsequent relapse vulnerability.

### Relapse-associated NAc gene expression

**Fig. 5:**
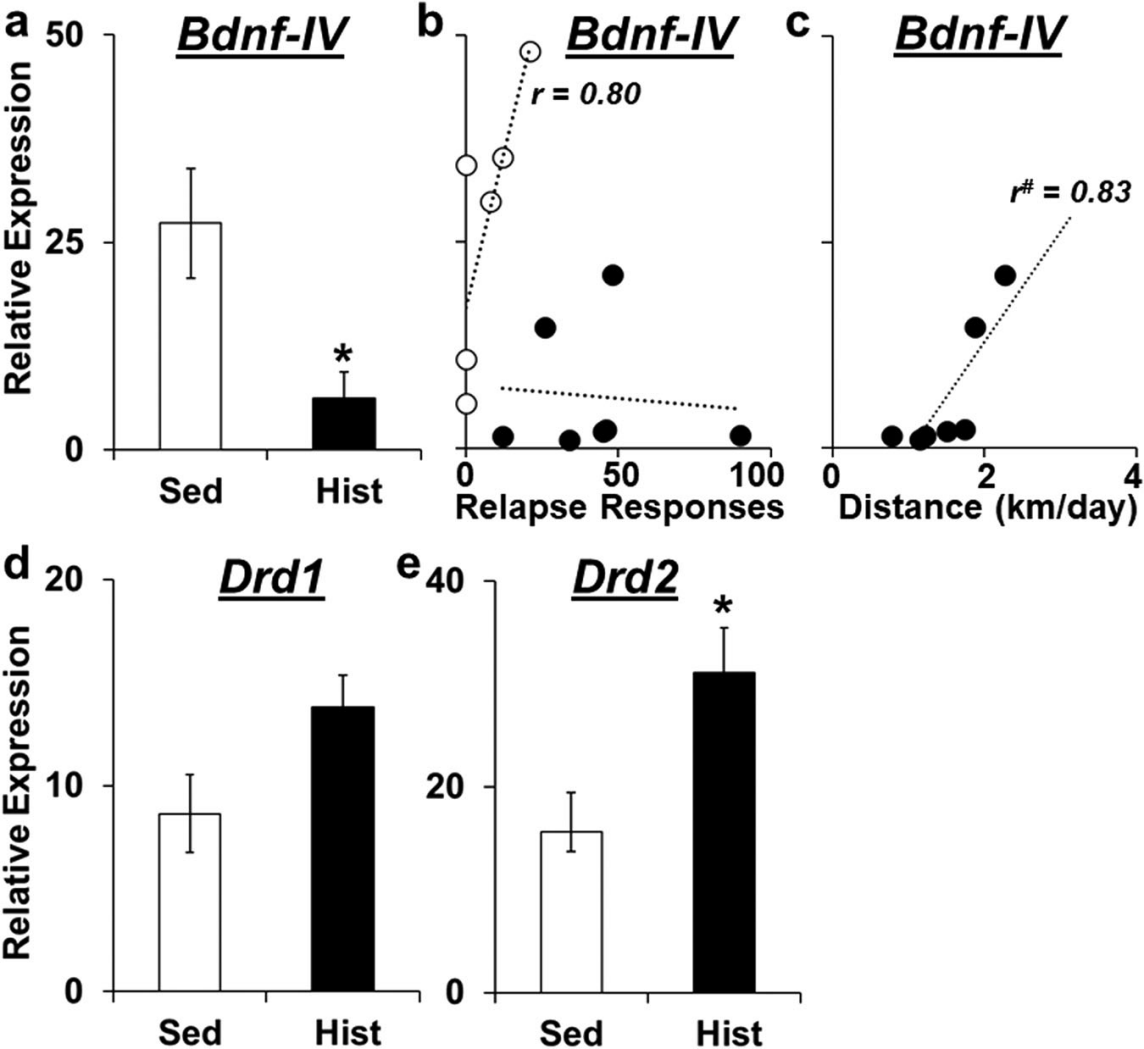
Effects of discontinued high-access exercise on relapse-associated gene expression. Relative *Bdnf-IV* expression (± SEM) in the nucleus accumbens (NAc) for sedentary (Sed) rats and rats in the history of exercise (Hist) group (**a**) and as a function of relapse responding for the sedentary and history of exercise groups (**b**) and within the history of exercise group as a function of average daily distance run over 2-week high-access exercise period (**c**). The Pearson correlation co-efficient (*r*-value) for the relationship between *Bdnf-IV* and relapse responses (**b**) or distance run (**c**) is also included. Relative *Drd1* (**d**) and *Drd2* (**d**) expression (± SEM) in the NAc for sedentary rats and rats in the history of exercise group. *Significant effect of group (*P* < 0.05; **a**, **e**); ^#^Significant association between *Bdnf-IV* expression and daily distance run (*P* < 0.05; **c**).

NAc gene expression differed between rats with, and without, a history of high-access exercise following relapse testing. Specifically, there was a robust group difference for *Bdnf-IV* expression (*t*_11_ = 3.0, *P* < 0.01) with the history of exercise group having markedly lower expression than the sedentary group (Fig. 5a). NAc *Bdnf-IV* expression also tended to positively correlate with relapse responses in the sedentary group (Fig. 5b; *t*_6_ = 0.8, *P* = 0.057), and within the exercise group, average distance run was positively associated with *Bdnf-IV* expression (*t*_7_ = 0.83, *P* < 0.05; Fig. 5c). *Drd1* expression tended to be higher in rats with, versus without, a history of exercise (Fig. 5d; *P* = 0.057). *Drd2* expression was significantly higher in the history of exercise group compared to the sedentary group (Fig. 5e; *t*_11_ = 2.3, *P* < 0.05). However, the associations between distance run and gene expression were non-significant for both groups for both *Drd1* and *Drd2*. There were also no group differences for *Drd3* or *Opmr1*. Thus, following relapse testing, rats with, versus without, a history of high-access exercise had decreased *Bdnf-IV* expression and increased *Drd2* expression, and tended to have increased *Drd1* expression. Levels of prior running also positively associated with NAc *Bdnf-IV* expression.

## Discussion

The goal of this study was to determine whether high-access exercise prior to and during early adolescence enhances later vulnerability to opioid use and OUD using rat models. Consistent with our hypothesis, rats with, versus without, a history of high-access exercise showed greater active-lever discrimination during acquisition, were more motivated to obtain fentanyl following acquisition, and had an enhanced sensitivity to the reinstating effects of fentanyl-associated cues following extended-access self-administration and protracted abstinence (14 days). In contrast, sedentary rats had higher overall responses during acquisition and higher inactive responses during extended-access self-administration as compared to history of exercise rats. Molecular markers of vulnerability to opioid use/relapse were also differentially expressed in the NAc of rats with, versus without, a history of high-access exercise following relapse testing with particularly robust differences observed for *Bdnf-IV* expression. Together, these findings show that even a brief period of high-access exercise prior to and throughout early adolescence can enhance sensitivity to the reinforcing and cue-induced reinstating effects of fentanyl and indicate that the enhanced vulnerability observed in middle- and high-school athletes could be due to the discontinuation of high-access exercise.

The idea that exercise could enhance vulnerability to opioid use and OUD is contrary to a large body of evidence in humans and animal models showing that exercise protects against drug use and addiction for multiple drugs, including opioids [13, 14, 49–52]. In fact, we and others have suggested exercise as a promising non-pharmacological intervention for addiction since, by activating the same reward pathway as addictive drugs, it can be used to normalize brain circuits disrupted in substance use disorder thereby decreasing drug-craving and relapse [13, 14, 49–52]. By this same reasoning, however, we proposed that, like addictive drugs, certain exercise conditions may “prime” the reward pathway thereby enhancing rather than decreasing vulnerability to addiction [13, 14]. Here, we focused on the potential for discontinued high-access exercise to enhance vulnerability to opioid use and OUD based on previous work showing that high-access exercise induces similar molecular changes in the reward pathway as addictive drugs [21–23, 53], and when discontinued, enhances the development of conditioned place preference induced by cocaine or morphine, the acquisition of methamphetamine self-administration, and motivation to obtain methamphetamine following extended-access self-administration [9–12]. Our findings extend this previous work to show that the discontinuation of high-access exercise enhances the reinforcing and cue-induced reinstating effects of opioids. It is notable that in the previous study with methamphetamine [10], continued access to exercise at a lower level (18–23 h/day) not only prevented the enhanced vulnerability, but as with previous findings [13, 14], continued exercise reduced vulnerability during acquisition and extended-access self-administration below that observed in sedentary controls. This finding is intriguing because it suggests that the enhanced vulnerability observed in middle- and high-school athletes could be prevented by continued exercise using a modified, lower-intensity, schedule. Future studies are necessary to address this possibility.

While discontinued high-access exercise did not impact rates or percent group acquisition, it did enhance active-lever discrimination during acquisition and PR responding for fentanyl following acquisition; levels of running during the high-access exercise period were also predictive of subsequent motivation for fentanyl. Together these findings indicate that a history of high-access exercise enhanced the reinforcing effects of fentanyl, which would presumably translate to an enhanced vulnerability to opioid use [54]. We observed similarly enhanced vulnerability in rats with, versus without, a history of exercise during relapse testing indicating that the discontinuation of high-access exercise persistently impacts vulnerability to both drug use and the development of key features of addiction, such as vulnerability to relapse.

In contrast to our results showing greater PR responding and vulnerability during reinstatement testing in discontinued exercise versus sedentary rats, discontinued exercise rats had lower overall responses during acquisition and lower inactive-lever responses during extended-access self-administration. While the findings during acquisition could reflect differential learning, rates of acquisition, or locomotor activation, the effects during extended-access likely reflect differential locomotor effects considering that acquisition of self-administration and the learning of lever contingencies had already occurred, and suggest that rats with a history of high-access exercise had a reduced sensitivity to the locomotor activating effects of fentanyl. This interpretation is also consistent previous work showing that discontinued high-access exercise protects against the development of sensitization to the locomotor activating effects of cocaine [55] and with numerous studies showing that the locomotor response to opioids (morphine, DAMGO [56, 57]); and other addictive drugs (cocaine; [19]) is decreased following moderate and high-access exercise. Moderate exercise (30 min, treadmill running) has also been reported to offset morphine withdrawal-induced increases in locomotor activity [58]. These findings also demonstrate differential effects of discontinued high-access exercise on the locomotor versus reinforcing/reinstating effects of fentanyl and add to a growing body of work indicating that each of these effects are dissociable [59–61]. This is important considering that locomotor activation is still often used as a proxy for the reinforcing effects of addictive drugs (e.g., [58, 62, 63]).

We previously showed that moderate exercise that decreased opioid self-administration increased *Bdnf-IV* expression and decreased *Oprm1* and *Drd1/2/3* expression in the NAc [33]. In this study where exercise enhanced, rather than decreased, vulnerability to opioid use/relapse, we observed opposite changes in the expression of *Bdnf-IV*, *Drd1*, and *Drd2* in the NAc such that rats with, versus without, a history of high-access exercise had markedly decreased *Bdnf-IV* expression and increased *Drd2* expression; they also tended to have increased *Drd1* expression. No effects were found for *Drd3* or *Oprm1* which could reflect a difference between fentanyl and heroin, aerobic and resistance exercise, or cue-induced reinstatement versus short-access intake. Future research is necessary to distinguish between each of these factors. Exercise markedly decreased *Bdnf-IV*, yet low *Bdnf-IV* expression in sedentary controls tended to predict low vulnerability during relapse testing. Higher levels of running in the exercise group were also predictive of higher *Bdnf-IV* expression following relapse testing. We predicted the direction of change by discontinued high-access exercise based on our previous findings with exercise [33], and it is notable that the overall group effect tracks with the efficacy of exercise to decrease (increased *Bdnf-IV*) or increase (decreased *Bdnf-IV*) vulnerability to opioid use/relapse. It is also consistent with findings showing that knockout of BDNF’s receptor, TrkB, from D_1_R-expressing neurons in the NAc enhances morphine reward [64]. None-the-less, the correlational findings within the sedentary controls are more consistent with the larger literature which indicates that enhanced *Bdnf-IV* expression and BDNF-Trkb signaling positively associates with incubated drug-craving [13, 45, 65]. One caveat is that this literature is mainly focused on cocaine, and while similar findings have been observed with opioids [66], there is also evidence indicating that the role of *Bdnf-IV* is different for opioids [27]. Future studies are necessary to determine whether *Bdnf-IV* plays a causal role in opioid use/relapse.

### Summary and translational implications

Our current findings indicate that the discontinuation of high-access exercise, which can also occur in human athletes following injury, enhances vulnerability to opioid use and relapse. Other breaks in regular high-intensity exercise occur in middle- and high-school athletes, but injury may be a special case since it is paired with access to opioids (e.g., via prescription). The translational implication of these findings is that awareness and intervention efforts should be expanded to include a consideration of the sensitizing effects of discontinued high-intensity exercise. For example, given that continued exercise, whether low- or high-access or aerobic or resistance, reduces vulnerability to opioid use in animal models [13, 14], it may be possible to prevent the enhanced risk observed in adolescent and young adult athletes using modified exercise interventions (lower intensity, resistance exercise). Future research is necessary to test this possibility. Future research is also necessary to determine whether similar effects occur in females. This is important considering that sex/gender differences are difficult to discern from the human contact sports-participation studies since males have been over-represented.
